# Latino and Black smokers in the Health and Retirement Study are more likely to quit: the role of light smoking

**DOI:** 10.1186/s12971-016-0090-y

**Published:** 2016-07-18

**Authors:** Frank C. Bandiera, Shervin Assari, Jennifer Livaudais-Toman, Eliseo J. Pérez-Stable

**Affiliations:** University of Texas School of Public Health, Dallas Regional Campus, 6011 Harry Hines Blvd., V8.112, Dallas, TX 75390-9128 USA; University of Texas Southwestern Harold C. Simmons Cancer Center, 6011 Harry Hines Blvd., V8.112, Dallas, TX 75390-9128 USA; Department of Psychiatry, School of Medicine, University of Michigan, 4250 Plymouth Rd, Ann Arbor, MI 48109 USA; Center for Research on Ethnicity, Culture and Health, School of Public Health, University of Michigan, 4250 Plymouth Rd, Ann Arbor, MI 48109 USA; Division of General Internal Medicine, University of California, 3333 California Street, San Francisco, CA 94143 USA; National Institute of Minority Health and Health Disparities, National Institutes of Health, 6707 Democracy Boulevard, Suite 800, Bethesda, MD 20892-5465 USA

**Keywords:** Latinos, Smoking cessation, Older persons

## Abstract

**Background:**

Older persons are more vulnerable to tobacco mortality and less likely to make quit attempts. Less is known, however, about the role of race and ethnicity on quit rates in the U.S. Using a nationally representative data source of older adults in U.S., we aimed to study racial and ethnic differences in smoking cessation rates.

**Methods:**

We used data from all waves of the Health and Retirement Study (HRS) between 1992-2012. The HRS is a longitudinal nationally representative survey of adults over the age of 50 in the United States. We followed current smokers at baseline (year 1992) until time to first quit. Race/ethnicity was the main predictor; gender, age, education, marital status, count of chronic medical conditions, depressive symptoms, and drinking at baseline were control variables. Cox regression was used for analysis of time to quit.

**Results:**

Hazard ratios of quitting during the first ten (Hazard ratio = 1.51, *p* < 0.05) and 20 years (Hazard ratio = 1.46, *p* < 0.05) were larger for Latinos over the age of 50 compared to Whites. In addition, hazard ratios of quitting during the first 20 years (Hazard ratio = 1.19, *p* < 0.05) were larger for Blacks over the age of 50 compared to Whites. These findings were partially explained by cigarette consumption intensity, such that Latinos were lighter smokers and therefore more likely to quit than Whites.

**Conclusion:**

Latinos and Blacks were more likely than Whites to quit smoking cigarettes within 20 years. However, this finding may be explained by cigarette consumption intensity.

## Introduction

Smoking has been related to many chronic diseases ranging from cancer, to heart disease, to stroke and others [1]. Although smoking onset usually occurs during youth,[1] smoking-related diseases do not typically manifest themselves until older age [1]. Although most smokers report that they would like to quit smoking completely [2], older smokers who have not successfully quit at younger ages are less likely to make quit attempts than younger smokers [3]. Thus, it is important to study older smokers given that most chronic diseases manifest in older age, and those who continue to smoke are less likely to make quit attempts. Studying cigarette smokers who are older is also important because older cohorts began smoking when prevalence rates were higher [4].

There are also race/ethnic differences in smoking rates, with 19.4 % of non-Latino Whites (hence Whites), 18.3 % of non-Latino Blacks (hence Blacks), and 12.1 % of Latinos being smokers in 2013 [5]. However, despite a lower rate of smoking, Black men have higher rates of tobacco-related illness (e.g., lung cancer) than Whites [6]. Studies reporting successful quit attempts by race/ethnicity have reported mixed findings in samples of adults of all ages. Further, in a review of the literature, Weinberger notes that little attention has been paid to Latinos [7]. Data in persons aged ≥18 years from the 2010 National Health and Interview Surveys (NHIS), which is a nationally representative sample of the U.S. population, suggest that Black smokers are more interested in quitting and making a quit attempt in the past year than White and Latino smokers [3]. However, findings from the same study indicated that Black smokers were less likely to quit in the past year compared to White smokers [3] and that Latino smokers were more likely to quit in the past year than White smokers [3]. Other studies among adults ages ≥18 from local non-national studies find no differences by race/ethnicity in quitting smoking [8, 9]. To our knowledge no studies have evaluated racial/ethnic differences in quitting smoking in older persons. Given that older persons are more likely to have tobacco-related morbidity and less likely to make quit attempts and given mixed findings by race/ethnicity, the purpose of the current study was to study racial/ethnic differences in smoking cessation rates in a nationally representative longitudinal sample.

## Methods

### Health and retirement study

We used longitudinal data from all waves of the Health and Retirement Study (HRS) between 1992 and 2012. The HRS is a longitudinal survey of a nationally representative sample of adults born in the U.S. between the years 1931 and 1941 and between the ages of 50 to 60 years at baseline interview. The HRS is widely recognized as the premier source of data on aging in the U.S. For the current study, baseline measures were collected in 1992; we evaluated 10-year follow-up measures in these participants in 2002 and 20-year follow-up measures in 2012. All participants provided written consent, and the study protocol was approved by the University of Michigan, Institutional Review Board (IRB).

The HRS sampling involves a multi-stage area probability design with geographic stratification and clustering. To ensure a large enough representation of minorities, HRS oversampled Black and Latino households at about twice the rate of Whites. The study produces sample weights that can be applied analytically in order to account for the differential probability of selection into the study and differential non-response. The follow-up rate is around 90 %. Our sample consisted of current smokers at baseline (in 1992).

### Smoking behavior

Participants were asked if they had ever smoked 100 or more cigarettes in their lifetime and if “yes”, this defined ever smokers. Participants were then asked if they smoked now (at the time of the survey) to define current smokers, as used in previous HRS studies [10–12]. The current study only included participants who were current smokers at baseline. The average number of cigarettes smoked per day was also ascertained Cessation at each follow-up survey was determined by a “no” response to the question “are you a smoker now?”

### Demographics, alcohol use, depression and chronic medical conditions

All demographic measures came from the baseline survey. Race/ethnicity was categorized into three mutually exclusive groups of White, Black, and Latino. Gender, age in years, marital status (married, formerly married, never married) and years of education (high school or more) were recorded [13].

Alcohol use was measured by average number of drinks per week [13]. Depressive symptoms were assessed at baseline by the eight item short-form Center for Epidemiologic Studies Depression (CES-D) scale. The items consisted of having the following experiences during the past week: *felt depressed, felt everything was an effort, sleep was restless, was happy, felt lonely, enjoyed life, felt sad, and could not get going*. Response options for each item were “none of the time, some of the time, most of the time, or all of the time” and were coded as “Yes” (if some, most, or all of the time) or “No” (if none of the time) and corresponded with the scores of 1 or 0, respectively, except for two items (*was happy and enjoyed life*) that were scored 0 (if none, some or all of the time) or 1 (if most of the time). A dichotomous version of depressive symptoms was created with the pre-established cut-off score of ≥4 to define significant symptoms. A count of six reported chronic medical conditions related to tobacco use was created and included diabetes mellitus, heart disease, emphysema or asthma, stroke, cancer and hypertension.

### Statistical analyses

Data were downloaded from the HRS website, Institute for Social Research (ISR), University of Michigan (http://hrsonline.isr.umich.edu/). Due to the complex sample design, Stata 13 was used for data analysis. Taylor series linearization was used for estimation of standard errors. Kaplan Meier curves were used to explore how smoking cessation rates over time varied based on race/ethnicity and covariates.

Cox regression was used to explore racial/ethnic differences in smoking cessation rates, after adjusting for all covariates including gender, age, marital status, chronic medical conditions, education level, alcohol consumption, and depressive symptoms. All of these covariates were measured at baseline. The first set of models were not adjusted for number of cigarettes smoked per day while the subsequent set of models were adjusted for number of cigarettes smoked per day.

We explored differences in smoking cessation rates at 10- and 20-year follow-up. For the Cox regression at ten years, follow-up time was calculated as the time from baseline to event (smoking cessation) or the time from baseline to the end of the ten years (2002) study for those who had not stopped smoking but were still participating. The latter were considered censored at ten years. Participants who had never stopped smoking but died or left the study before the 10-year (2002) interview were considered censored at the time of their departure from the study or death. Similarly, for the Cox regression at 20 years, follow-up time was calculated as the time from baseline to event (smoking cessation) or the time from baseline to the end of the 20-year (2012) study for those who had not stopped smoking but were still participating. The latter were considered censored at 20 years. Participants who had never stopped smoking but died or left the study before the 20-year (2012) interview were considered censored at the time of their departure from the study or death. Hazard Ratios (HR) with 95 % CI were reported and HRs greater than 1 indicates increased chance of quitting.

## Results

### Description of the sample by race/ethnicity

Approximately 50 % of participants were women; 569 were Black, 244 were Latino, and 2,156 were White; approximately 87 % were ages 50–59 years and 13 % were 60+. There was no difference in age between Whites, Blacks and Latinos, *p* < 0.05. There were more White women (51 %) than Black (44 %) and Latino women (44 %), *p* < 0.05. Whites (71 %) were more likely to have obtained a high school diploma or greater than Blacks (54 %) and Latinos (34 %), *p* < 0.05. Whites (23) on average smoked more cigarettes per day than Blacks (13) and Latinos (16), *p* < 0.05. Table 1 describes demographics, depression, drinking, and quit rates by race/ethnicity.

**Table 1:** Descriptive statistics of 2969 Black, Latino and White Older Adults (age >50 at baseline) who were current smokers at baseline (year 1992)

	Black (*N* = 569)	Latino (*N* = 244)	White (*N* = 2,156)	Total	*p*-value
	%	%	%	%	
Age group (range 50–74)					
50–59 years	86.5	89.8	87.3	87.4	0.554
60+ years	13.5	10.2	12.7	12.6	
Gender					
Women	44.2	43.6	51.3	49.9	0.008>
Marital status					
Married	46.2	60.4	72.6	68.7	<0.001
Formerly married	45.2	34.7	25.0	28.1	
Never married	8.6	4.9	2.3	3.2	
Education					
Low (less than high school)	45.8	65.9	28.8	33.1	<0.001
High (high school or greater)	54.2	34.1	71.2	66.9	
Chronic medical condition count^a^ (range 0–6) (mean, SE)	1.1 (0.06)	0.8 (0.07)	0.8 (0.02)	0.8 (0.02)	<0.001
Depression: CES-D 8 item scale (score ≥4)	36.6	43.5	28.6	30.4	<0.001
Alcohol: drinks per day					
0	36.1	39.2	31.6	32.6	0.044
1-2/day	55.6	49.5	58.1	57.3	
> 2/day	8.3	11.3	10.3	10.1	
Average number of cigarettes smoked per day (mean, SE)	13.4 (0.39)	15.8 (1.24)	22.6 (0.36)	21.0 (0.29)	<0.001
Quit within 10 years (restricted to those still being followed in 2002)					
Yes	46.8	52.0	45.9	46.4	0.438
Quit within 20 years (restricted to those still being followed in 2012)					
Yes	64.0	65.3	64.4	64.4	0.974

*CES-D* Center for Epidemiological Studies Depression (CESD); 8 –item version of scale was used; scores of four or greater considered depressed

^a^Count of chronic medical conditions includes: diabetes, heart disease, emphysema/asthma, stroke, cancer, and hypertension

### Cox regression

In Cox regression models that were unadjusted for number of cigarettes smoked per day, compared to Whites the hazard ratio of quitting during the first 10 years was larger for Latinos (HR = 1.51, 95 % CI = 1.18–1.94) but not for Blacks (Table 2), and the hazard ratio of quitting during the first 20 years was larger for both Latinos (HR = 1.46, 95 % CI = 1.16–1.84) and Blacks (HR = 1.19, 95 % CI = 1.01–1.44) (Table 2). However, when models were adjusted for cigarettes per day, there were no racial/ethnic differences in quitting within ten years or within 20 years (Table 3).

**Table 2:** Summary of Cox model among 2969 Black, Latino and White Older Adults (age >50 at baseline) who were current smokers at *baseline* not adjusting for cigarettes per day (year 1992)

	Cessation at 10 years		Cessation at 20 years	
	HR(SE)	95 % CI	HR(SE)	95 % CI
Race/ethnicity				
White (referent)				
Black	1.19 (0.10)	0.99–1.41	1.19 (0.11)*	1.01–1.44
Latino	1.51 (0.19)**	1.18–1.94	1.46 (0.17)**	1.16–1.84
Gender				
Men (referent)				
Women	0.93 (0.05)	0.83–1.04	0.93 (0.05)	0.93–1.04
Age group at baseline				
50–59 years (referent)				
60+ years	1.31 (0.14)*	1.06–1.62	1.21 (0.11)*	1.01–1.46
Education at baseline				
< High school (referent)				
High school or more	1.26 (0.11)*	1.06–1.51	1.19 (0.10)*	1.01–1.40
Marital Status at baseline				
Married (referent)				
Formerly married	0.95 (0.09)	0.79–1.15	0.94 (0.09)	0.78–1.14
Never Married	0.60 (0.16)	0.36–1.01	0.70 (0.15)	0.46–1.08
Count of chronic medical conditions (continuous)	1.05 (0.03)	0.99–1.12	1.05 (0.03)*	1.01–1.11
Drinking Frequency per day at baseline				
None (referent)				
1-2 drinks per day	0.94 (0.06)	0.83–1.07	0.99 (0.06)	0.88–1.10
> 2 drinks per day	0.58 (0.08)***	0.44–0.77	0.64 (0.08)**	0.50–0.83
Depressive Symptoms: CESD-8 score (range 0–8)				
Score <4 (referent)				
Score ≥4	1.12 (0.09)	0.96–1.31	1.11 (0.06)	0.99–1.24

*P < 0.05 ** *P* < 0.01 ***P < 0.001

**Table 3:** Summary of Cox model among 2969 Black, Latino and White Older Adults (age >50 at baseline) who were current smokers at *baseline* adjusting for cigarettes per day (year 1992)

	Cessation 10 years		Cessation 20 years	
	HR(SE)	95 % CI	HR(SE)	95 % CI
Race/ethnicity				
White (referent)				
Black	0.95 (0.09)	0.78–1.16	0.97 (0.10)	0.80–1.19
Latino	1.24 (0.17)	0.95–1.63	1.21 (0.15)	0.94–1.56
Gender				
Men (referent)				
Women	0.84 (0.05)**	0.74–0.94	0.85 (0.05)**	0.75–0.96
Age group at baseline				
50–59 years (referent)				
60+ years	1.28 (0.13)*	1.04–1.57	1.19 (0.11)	0.99–1.43
Education at baseline				
< High school (referent)				
High school or more	1.23 (0.11)*	1.03–1.46	1.16 (0.10)	0.98–1.37
Marital Status at baseline				
Married (referent)				
Formerly married	0.95 (0.09)	0.79–1.14	0.94 (0.09)	0.78–1.14
Never Married	0.62 (0.15)	0.38–1.02	0.73 (0.15)	0.49–1.10
Count of chronic medical conditions (continuous)	1.08 (0.03)*	1.01–1.15	1.08 (0.03)**	1.02–1.13
Average number of cigarettes smoked per day (continuous)	0.98 (0.01)***	0.97–0.98	0.98 (0.01)***	0.97–0.98
Drinking Frequency per day at baseline				
None (referent)				
1-2 drinks per day	0.95 (0.06)	0.84–1.08	0.99 (0.05)	0.89–1.11
> 2 drinks per day	0.66 (0.08)**	0.51–0.85	0.71 (0.08)**	0.57–0.90
Depressive Symptoms: CESD-8 score (range 0–8)				
Score <4 (referent)				
Score ≥4	1.14 (0.09)	0.98–1.34	1.13 (0.06)*	1.01–1.26

*P < 0.05 ** *P* < 0.01 ***P < 0.001

## Discussion

In our study we found that among current smokers, Latinos and Blacks were more likely to quit smoking than Whites within 20 years of follow-up. However, cigarette smoking intensity, as measured by number of cigarettes smoked per day, partially explained these findings. This is important because as the prevalence of cigarette smoking keeps declining and persons become lighter smokers, then it would be easier to quit regardless of race/ethnicity. Further, for interventionists and public health efforts, it might be easier to get lighter smokers to quit than heavier smokers. Our study adds to the current literature by considering an older age group that typically engages in fewer quit attempts [2] and is more vulnerable to tobacco mortality. Our paper also incorporates longitudinal data which addresses some of the limitations from earlier published findings from the NHIS [2], using cross-sectional data. Our study also adds to the current literature by identifying Latinos as being more likely to quit smoking within 10 and 20 years of baseline, which might have implications for better health outcomes and longevity [14]. We also identified an important mechanism, cigarette smoking intensity, which appears to contribute to racial/ethnic differences in smoking cessation.

One reason why Latinos may be more likely to quit smoking is because they tend to be lighter or more intermittent smokers [15]. Thus, they may be less dependent on nicotine and more easily able to quit. Indeed, in our study, we did find that cigarette smoking intensity partially explained the significant findings among Latinos. On the other hand, there were no differences between Blacks and Whites at ten years, consistent with findings from smaller non-representative samples that have found no significant differences [8, 9]. However, at 20 years, Blacks were more likely to quit than Whites and this finding was also explained by lower number of cigarettes per day on average. Our results might also help to explain why mortality rates for Blacks and Latinos have continued to decrease in the past 15 years [16]. Indeed, the Centers for Disease Control and Prevention attribute the better health in Latinos in part to lower smoking rates [17].

The strengths of this study include that it is a nationally representative sample and a large number of older adults were surveyed. Another strength is the longitudinal design with long-term follow-up. We were also able to control for a number of covariates and used a standard measure of smoking cessation. Limitations include that this analysis only applied to persons over age 50 years from a particular birth cohort and as tobacco control policies have been implemented over time, this may affect the results we would in subsequent cohorts of older smokers. In addition, smoking data were self-reported and were not validated with a biochemical test. Also, we were unable explore racial/ethnic differences in smoking cessation rates for ever smokers who had quit prior to baseline. Another limitation is that depressive symptoms and alcohol use were measured at baseline and it is possible that these covariates changed over time. Nevertheless, these are important covariates to consider since they may influence smoking cessation.

In conclusion we found that among current smokers at baseline, Latinos were more likely to quit smoking cigarettes within ten and 20 years of follow-up and this finding was explained in part by cigarette consumption. Similarly, Blacks were more likely to quit within 20 years and this finding was also explained by cigarette consumption. Future studies should explore whether racial/ethnic differences in smoking cessation vary according age group. Future studies should also consider how lighter smoking may lead to increased smoking cessation as it may be easier for people who are not as dependent on smoking to quit smoking.
